# Cocoa Flavonoid-Enriched Diet Modulates Systemic and Intestinal Immunoglobulin Synthesis in Adult Lewis Rats

**DOI:** 10.3390/nu5083272

**Published:** 2013-08-19

**Authors:** Malen Massot-Cladera, Àngels Franch, Cristina Castellote, Margarida Castell, Francisco J. Pérez-Cano

**Affiliations:** Departament de Fisiologia, Facultat de Farmàcia, Universitat de Barcelona, Institut de Recerca en Nutrició i Seguretat Alimentària (INSA-UB), Barcelona 08028, Spain; E-Mails: malen.massot@ub.edu (M.M.-C.); angelsfranch@ub.edu (À.F.); cristinacastellote@ub.edu (C.C.); margaridacastell@ub.edu (M.C.)

**Keywords:** cocoa polyphenols, immune system, immunoglobulins

## Abstract

Previous studies have reported that a diet containing 10% cocoa, a rich source of flavonoids, has immunomodulatory effects on rats and, among others effects, is able to attenuate the immunoglobulin (Ig) synthesis in both systemic and intestinal compartments. The purpose of the present study was focused on investigating whether these effects were attributed exclusively to the flavonoid content or to other compounds present in cocoa. To this end, eight-week-old Lewis rats were fed, for two weeks, either a standard diet or three isoenergetic diets containing increasing proportions of cocoa flavonoids from different sources: one with 0.2% polyphenols from conventional defatted cocoa, and two others with 0.4% and 0.8% polyphenols, respectively, from non-fermented cocoa. Diet intake and body weight were monitored and fecal samples were obtained throughout the study to determine fecal pH, IgA, bacteria proportions, and IgA-coated bacteria. Moreover, IgG and IgM concentrations in serum samples collected during the study were quantified. At the end of the dietary intervention no clear changes of serum IgG or IgM concentrations were quantified, showing few effects of cocoa polyphenol diets at the systemic level. However, in the intestine, all cocoa polyphenol-enriched diets attenuated the age-related increase of both fecal IgA and IgA-coated bacteria, as well as the proportion of bacteria in feces. As these effects were not dependent on the dose of polyphenol present in the diets, other compounds and/or the precise polyphenol composition present in cocoa raw material used for the diets could be key factors in this effect.

## 1. Introduction

Cocoa, obtained from dried and fermented seeds derived from *Theobroma cacao*, was consumed as early as 1100 B.C. by ancient Mesoamerican civilizations [1]. In fact, the Olmecs, and eventually the Mayans and Aztecs, consumed food and beverages produced from cocoa, which was considered a divine beverage with medicinal properties [2]. Although health benefits of cocoa were already known by these ancient civilizations, it was not until recently that cocoa gained recognition because of its high content of fiber and polyphenols, particularly flavonoids. Cocoa powder mainly contains flavanols such as (−)-epicatechin and (+)-catechin (5%–10% of total polyphenols) as well as procyanidins (≥90%), the oligomers derived from these monomers [3,4,5,6,7,8,9,10,11]. Other minor polyphenols have been identified in cocoa, e.g., quercetin, isoquercitrin (quercetin 3-*O*-glucoside), quercetin 3-*O*-arabinose, hyperoside (quercetin 3-*O*-galactoside), naringenin, luteolin, apigenin, and others [12].

In addition to the antioxidant capacity of cocoa, its interaction with the immune system *in vitro* [13,14,15,16] and *in vivo* has been reported. Regarding *in vivo* effects, previous studies have demonstrated that a dietary intervention with cocoa was capable of modulating the serum IgA, IgM and IgG concentrations as well as the intestinal IgA and IgM contents in young rats [17,18,19]. In addition, a 10% cocoa diet was able to modify the composition and functionality of several lymphoid tissues [17,20,21,22] including the gut-associated lymphoid tissue (GALT) [20,21]. Cocoa intake modified, at the same time, the fecal microbiota composition and its crosstalk with the immune system by means of the modulation of the toll-like receptors’ (TLR) gene expression in the colon [19].

As occurs in other plant-derived foods, the phenolic content of cocoa-derived products is largely dependent upon the cultivar, origin, agricultural and postharvest practices, and the processing [11,23,24]. Until the cocoa liquor is obtained, the cocoa seed has to be subjected to different procedures (specifically fermentation, alkalinization or *Dutching* and roasting processes) which lead to a considerable loss of cocoa polyphenols [9,11,24]. On account of the beneficial effects and the poor bioavailability of polyphenols, developing a new flavonoid-enriched cocoa powder has become a subject of great interest. In order to achieve this, control of the processing steps as well as some modifications of its habitual processing procedure have been proposed to keep the maximum amount of cocoa polyphenols [24]. To this end, Naturex S.A. developed CocoaPure, a new flavonoid-enriched cocoa powder from unfermented, blanch-treated, and non-roasted cocoa beans [25].

In this regard, two types of cocoa extracts with a different proportion and composition of polyphenols have been used to ascertain whether the cocoa modulatory effects on the systemic and intestinal immunoglobulin synthesis observed in previous studies can be attributed exclusively to its flavonoid content.

## 2. Material and Methods

### 2.1. Animals and Diets

Twenty-four male Lewis rats (8 weeks old) were obtained from Harlan (Barcelona, Spain) and housed in cages under conditions of controlled temperature and humidity in a 12:12 light-dark cycle. After one week of acclimatization, the rats were randomly assigned to four dietary groups (*n* = 6/each): the reference group (REF) which was fed with the standard diet AIN-93M (Harlan, Barcelona, Spain); the cocoa group (PC0.2), which received chow containing 0.2% of polyphenols from conventional cocoa powder; and the PC0.4 and PC0.8 groups, receiving 0.4% and 0.8% of polyphenols, respectively, from non-fermented cocoa (CocoaPure). The diet lasted two weeks. Conventional natural Forastero cocoa containing 21.20 mg/g of polyphenols was provided by Nutrexpa S.L. CocoaPure containing 510 mg/g of polyphenols was provided by Naturex Spain S.L. The three cocoa diets were prepared from a basal mix diet and particular components, both supplied by Teklad Global Diets (Harlan, Indianapolis, IN, USA). The addition of 100 g/kg of conventional cocoa, 8.7 g/kg or 17.8 g/kg CocoaPure was established in order to obtain a final diet with 0.2%, 0.4% and 0.8% of polyphenols, respectively, and the same proportion of macronutrients as AIN-93M formula. (Table 1). The polyphenol characterization of PC0.2, PC0.4 and PC0.8 diets is also summarized in Table 1.

**Table 1:** Composition of experimental diets (g/kg diet). The three experimental diets provided similar amounts of proteins, lipids and carbohydrates to the reference diet (AIN-93M).

Components	REF (AIN-93M; g/kg)	PC0.2 (g/kg)	PC0.4 (g/kg)	PC0.8 (g/kg)
Cocoa powder	-	100	8.7	17.4
**Nutrients provided by the basal mix:**				
Casein	121.8	97.1	120.7	119.5
l-Cystine	1.8	1.4	1.8	1.8
Corn Starch	419.1	421.0	418.3	417.5
Maltodextrin	147.3	116.8	147.3	147.3
Sucrose	117.0	108.7	117.0	117.0
Soybean oil	40.0	26.2	39.4	38.9
Cellulose	50.0	24.5	48.3	46.5
Minerals	27.0	27.8	27.0	27.0
Vitamins	2.0	7.2	2.0	2.0
Choline bitartrate	2.5	2.0	2.5	2.5
*tert-*Butylhydroquine	0.008	0.006	0.008	0.008
**Nutrients provided by cocoa powder:**				
Protein	-	22	1.13	2.26
Carbohydrate	-	16	0.82	1.64
Lipid	-	11	0.56	1.12
Fiber (insoluble/soluble)	-	34 (25.5/8.5)	1.75 (1.31/0.44)	3.5 (2.63/0.88)
Total polyphenols *	-	2.12	4.44	8.88
Polyphenols provided by cocoa powder **:				
Catechin	-	0.073	0.048	0.097
Epicatechin	-	0.204	0.689	1.379
Isoquercetin	-	0.0053	n.d.	n.d.
Quercetin	-	0.0029	n.d.	n.d.
Procyanidin B_1_	-	n.d.	0.127	0.254
Procyanidin B_2_	-	0.167	0.356	0.713
Total procyanidins	-	n.d.	3.897	7.795

* The analytical method employed to estimate the total polyphenol compounds was the spectrophotometric method of Folin-Ciocalteu [26]. ** Polyphenol profile was identified and quantified using a reversed-phase high performance liquid chromatography (RP-HPLC) coupled to a diode array detector (DAD) (RP-HPLC-DAD) [27], and procyanidin B1 and B2 were quantified by normal-phase HPLC coupled to mass spectrometry (MS) [9] with fluorescence detection (n.d. means non determined).

Animals were given free access to water and chow. Body weight and food intake were monitored twice per week throughout the experiment. Studies were performed according to the criteria outlined by the Guide for the Care and Use of Laboratory Animals. Experimental procedures were reviewed and approved by the Ethical Committee for Animal Experimentation of the University of Barcelona.

### 2.2. Sample Collection and Processing

From the beginning of the diet until the end of the study, blood was collected through the saphenous vein and, after centrifugation, serum was separated and kept at −20 °C until ELISA quantification. Fecal samples were collected at the same time points and kept at −20 °C until analysis. Fecal homogenates for bacteria proportion and immunoglobulin-coating bacterial analysis (5% w/v) and ELISA IgA quantification (2% w/v), were obtained as previously described [19,20] and frozen at −20 °C until analysis.

### 2.3. Fecal pH Determination

Feces collected at the beginning and at the end of the nutritional intervention were used to determine fecal pH using a surface electrode (Crison Instruments, S.A., Barcelona, Spain).

### 2.4. Immunoglobulin Quantification in Serum and Feces (ELISA)

Fecal IgA, serum IgM and IgG concentrations were quantified by ELISA as previously described [28]. In brief, 96-well polystyrene plates (Nunc Maxisorp, Wiesbaden, Germany) were coated with anti-rat IgA, IgM or IgG mAbs (BD Biosciences, Heidelberg, Germany) (2 mg/mL in phosphate-buffered saline, PBS) and, after blocking with 1% bovine serum albumin (BSA, Sigma-Aldrich, Madrid, Spain) in PBS (PBS-BSA, 1 h, room temperature), appropriate diluted samples and standard dilutions (purified IgA, IgM or IgG, BD Biosciences) were added. After washing, biotin-conjugated anti-rat IgA, IgM or IgG mAbs (BD Biosciences) and subsequently, peroxidase-conjugated ExtrAvidin (Sigma-Aldrich) was added. An *o*-phenylenediamine dihydrochloride-H_2_O_2_ solution (Sigma-Aldrich) was used for the detection of bound peroxidase. 3 M H_2_SO_4_ was added to stop the reaction. Optical density (OD) was measured on a microtiter microplate photometer (Labsystems Multiskan, Helsinki, Finland) at 492 nm. Data were interpolated by means of Ascent v.2.6 software (Thermo Fisher Scientific, S.I.U., Barcelona, Spain) into the standard curves, and expressed as µg/mL in serum and as ng/mL in faecal samples.

### 2.5. Fecal Bacteria and IgA-Coating Bacterial Analysis

A volume of 35 µL of the fecal homogenates was diluted in 500 μL 1% (v/v) fetal bovine serum (FBS)/PBS and, after centrifugation (8000 *g*, 5 min, 4 °C), the resulting pellet was resuspended in 50 µL 1% (v/v) FBS/PBS containing fluorescein isothiocyanate (FITC)-anti-rat Ig antibodies (Abcam, Cambridge, UK). Each mixture was incubated for 30 min in the dark at room temperature. A non-stained mixture of each sample was used as control. Thereafter, suspensions were washed twice with PBS (8000 *g*, 5 min) and resuspended in 200 µL PBS until analysis. The labeled samples were mixed with 10 µL propidium iodide (PI, Sigma–Aldrich) (1 mg/mL) 15 min prior to flow cytometry (FCM) analysis in order to label total bacteria [19]. A FacsAria SORP sorter (BD, San José, CA, USA) was used. The FCM parameters were adjusted to obtain particle counts of 100–1000 events per second and the events were recorded for 60 s in list mode files. Data analysis was performed using the FlowJo v7.6.5 software (Tree Star, Inc., Ashland, OR, USA).

For bacteria proportion evaluation, a gate according to forward- and side-scattered light (FSC/SSC) was established on the basis of bacteria morphology from previous studies by using specific rRNA probes. The percentage of events stained with PI (by means of 610/20 nm fluorescence detectors) inside this gate is indicative of the proportion of bacteria present in the fecal sample. For IgA-coating bacteria, FITC-anti-rat Ig bound to bacteria were detected with the FITC fluorescence detectors (530/30 nm) and the IgA-coating bacteria proportion was expressed as positive FITC counts within the PI positive bacteria present in the above FSC/SSC morphology gate. Previously, we had demonstrated that Ig coated to bacteria was exclusively IgA [19].

### 2.6. Statistical Analysis

The software package SPSS 20.0 (SPSS, Inc., Chicago, IL, USA) was used for statistical analysis. Levene’s and Kolmogorov-Smirnov’s tests were applied to evaluate the equality of variance and normal distribution assays of the studied groups, respectively. The two-way analysis of variance followed by Bonferroni’s *post hoc* significance test was used when the assumptions of normality and equal variance were found in variables along time (*i.e.*, weight, chow intake, IgG). In the opposite case, non-parametric tests (Kruskall-Wallis and Mann-Whitney U rank-sum test) were used to assess significance (*i.e.*, IgM and IgA). Moreover, the non-parametric Wilcoxon test was used to compare matched groups at unique time point (*i.e.*, total bacteria at the end of the period). Significant differences were established at *P* values less than 0.05 when comparing two variables, and less than 0.0167 when comparing four variables (*i.e.*, four experimental diets).

## 3. Results

### 3.1. Body Weight and Chow Intake

**Figure 1:**
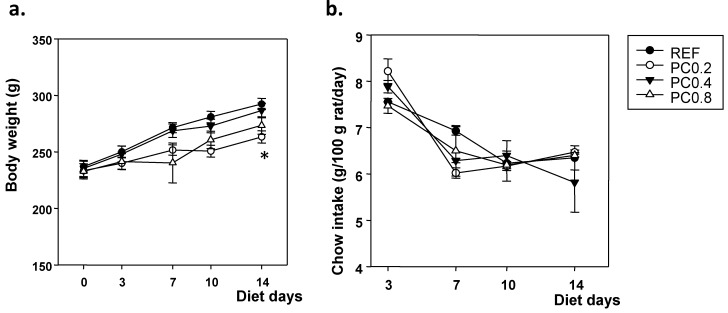
(**a**) Body weight and (**b**) chow intake (g/100 g rat/day) monitored during the period of dietary intervention with the cocoa polyphenol-enriched diets. Values are expressed as mean ± SEM (*n* = 6). Statistical differences: * *p* < 0.0167 *vs.* reference group (REF).

During the study, the body weight slope was similar between the groups with the exception of animals, which received the diet with a lower polyphenol content (PC0.2 group) who showed a lower increase than that of the reference rats, being statistically significant at the end of the study (Figure 1a). This effect was not related to a lower chow intake, as chow intake in all polyphenol-enriched groups and in the reference group ranged from 5 to 9 g/100 g/day, with no statistical differences between them (Figure 1b).

### 3.2. Serum IgG and IgM Concentrations

**Figure 2:**
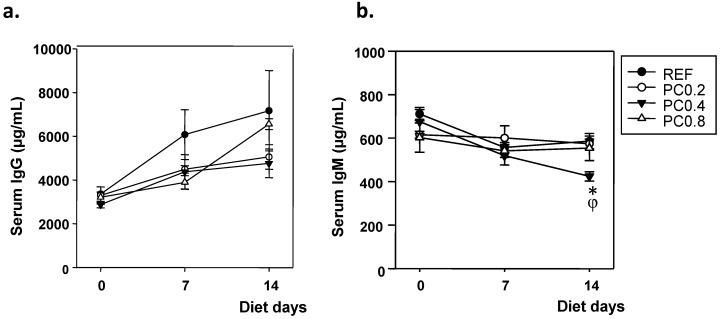
Effects of cocoa polyphenol-enriched diets on serum IgG (**a**) and IgM (**b**) concentrations at the beginning, and after one and two weeks of diet. Values are expressed as mean ± SEM (*n* = 6). Statistical differences: * *p* < 0.0167 *vs.* REF and φ *p* < 0.0167 *vs.* cocoa group (PC)0.2.

Serum concentrations of IgG and IgM from all experimental groups throughout the study are summarized in Figure 2. Although none of the cocoa polyphenol-enriched diets was able to significantly modify the serum IgG concentration, all dietary interventions had a tendency to lower the age-increasing pattern (Figure 2a). With regards to serum IgM antibodies, the animals fed with PC0.4 diet showed a significant decrease of IgM concentration two weeks after cocoa polyphenol intake began (Figure 2b) compared to those of the REF and PC0.2 groups (*p* < 0.0167).

### 3.3. Fecal IgA Concentration

**Figure 3:**
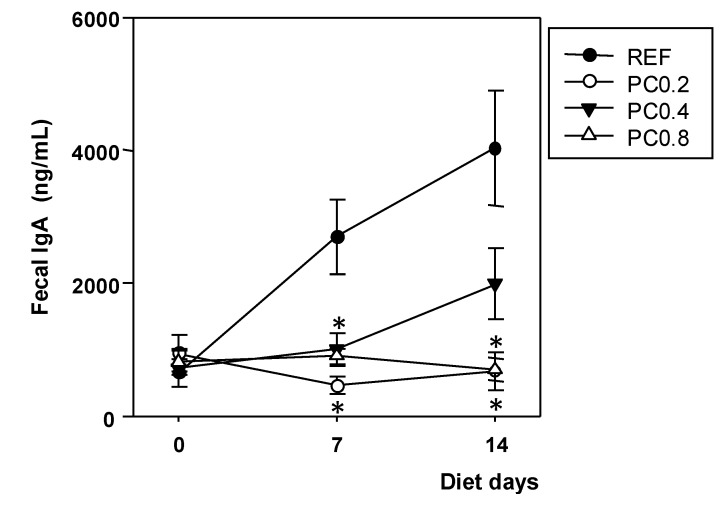
Effect of cocoa polyphenol-enriched diets on fecal IgA concentration at the beginning of the study and after one and two weeks of dietary intervention. Values are expressed as mean ± SEM (*n* = 6). Statistical differences: * *p* < 0.0167 *vs.* REF.

Intestinal IgA production was quantified in feces. The fecal IgA concentration increased about four-fold over the study period in REF rats. Overall, this age-dependent increase was avoided by all cocoa polyphenol-enriched diets. IgA concentrations were already lower than in the REF group one week after nutritional intervention in the PC0.2 and PC0.8 groups (*p* < 0.0167) and remained significant until the end of the study (Figure 3).

### 3.4. Total Bacteria Determination

**Figure 4:**
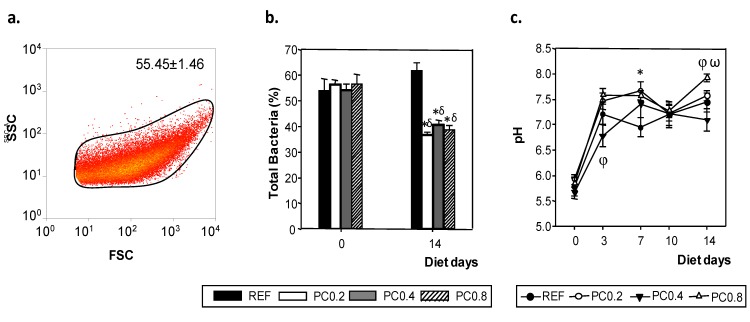
Representative biparametric cytogram showing the initial acquisition gate to select the bacterial population according to its forward- and side-scattered light (FSC/SSC) signal with the mean ± SEM of all groups at the baseline (**a**). Effect of cocoa polyphenol-enriched diets on total bacteria (**b**) and fecal pH (**c**) after two weeks of nutritional intervention. Results are expressed as mean ± SEM (*n* = 6). Statistical differences: δ *p* < 0.05 *vs.* initial values, * *p* < 0.0167 *vs.* REF, φ *p* < 0.0167 *vs.* PC0.2 and ω *p* < 0.0167 *vs.* PC0.4 in same day of study.

Fecal samples collected at the beginning of the diet and two weeks after the nutritional intervention allowed us to determine the percentage of total bacteria. For that, bacteria morphology was selected according to their FSC/SSC signal and by means of PI fluorescence detectors (Figure 4a). There was no difference in the percentage of total bacteria in any of the groups at the baseline (55.45% ± 1.46%). The REF group showed a significant age-related increase in this percentage, from ~55% at the beginning of the diet to ~62% two weeks later (*p* < 0.05). This pattern was avoided by all cocoa polyphenols-enriched diets regardless of dose (*p* *<* 0.0167) (Figure 4b).

Fecal samples collected weekly allowed fecal pH to be determined (Figure 4c). Although there were some punctual differences at Day 7 for the PC0.2 diet and at the end of the study for PC0.8, no significant changes due to the nutritional interventions of cocoa were observed and a similar pattern was seen for pH.

### 3.5. IgA-Coating Bacteria Analysis

**Figure 5:**
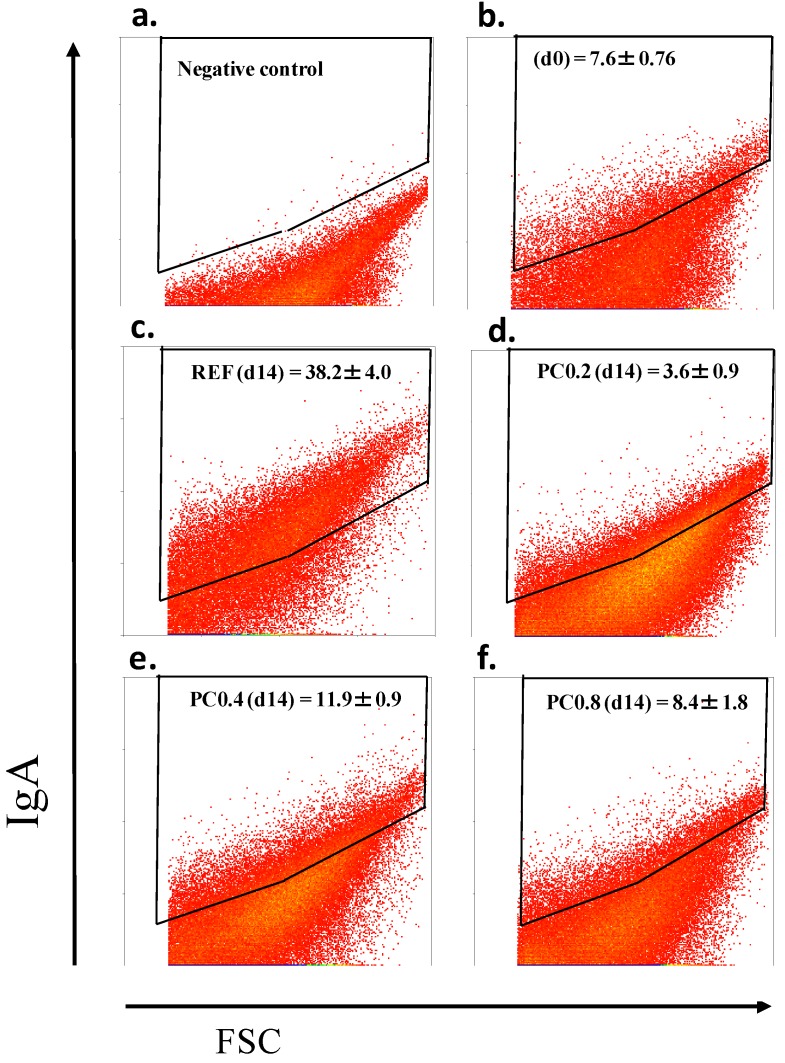
Effects of cocoa polyphenol-enriched diets on percentage of fecal bacteria coated with IgA before and after 2 weeks of nutritional intervention. Biparametric cytogram corresponding to a negative fecal control (**a**). It combines morphological parameters of the bacterial population (FSC/SSC) and fluorescence for IgA-fluorescein isothiocyanate (FITC) which allow the threshold for the positive region to be established. Cytogram, which represents the percentage of IgA-coating bacteria at baseline for all groups (**b**). Cytograms for IgA-coating bacteria detection for REF (**c**), PC0.2 (**d**), PC0.4 (**e**) and PC0.8 (**f**) groups at the end of the study. Results are expressed as mean ± SEM (*n* = 6).

IgA-coating bacteria were determined by flow cytometry using fecal samples collected before the start of the diet and at the end of the study (Figure 5). A non-stained sample was used as a negative control (Figure 5a). At the beginning, all groups showed a similar IgA-coating pattern (Figure 5b). The REF group showed an age-related increase in the percentage of IgA-coating bacteria from up to ~38% two weeks later (Figure 5c). This pattern was avoided by all cocoa polyphenol-enriched diets (*p* < 0.0167) (Figure 5d–f) with the down modulatory effect in animals that received the diet with a lower polyphenol content (PC0.2 group, *p* < 0.0167) (Figure 5d) being significantly higher.

## 4. Discussion

In previous studies, cocoa-enriched diets have demonstrated their immunomodulatory role on rats’ systemic and intestinal compartments. It is believed that this effect is mainly due to the polyphenols present in cocoa. The purpose of the present work was focused on investigating whether these effects are attributed exclusively to its polyphenol content or to other compounds present in cocoa. For that, Lewis rats were fed three cocoa diets formulated with two distinct cocoa raw materials differing in polyphenol dose and composition. The study lasted two weeks, because in previous studies, this period was enough to down-regulate intestinal IgA [20]. From the present results it emerges that all dietary interventions with cocoa attenuated the humoral intestinal immune response, demonstrating for the first time the immunomodulatory effect of non-fermented-polyphenolic-enriched cocoa. Although all dietary interventions with cocoa attenuated the humoral intestinal immune response, it was achieved in a polyphenol-dose-independent manner suggesting that other compounds present in cocoa powder also play a role.

Previous reports concerning the effect of cocoa on the immune system *in vivo* have been developed using 2%–10% cocoa-enriched diets formulated with defatted cocoa containing 20–30 mg/g of polyphenols [19,20,21]. In the present study we used a conventional cocoa powder with 2% of polyphenols to elaborate the PC0.2 diet. We added 100 g of cocoa powder to 900 g of basal mix and the resulting diet contained 0.2% polyphenols in addition to the proteins (2.2%), carbohydrates (1.6%), lipids (1.1%) and fiber (3.5%) provided by cocoa. The present work includes the study of the effect of CocoaPure, which is a cocoa powder obtained by a modification of conventional cocoa powder processing, thus avoiding the loss and keeping the high amount of polyphenols present in the cocoa seed [26]. In the elaboration of the CocoaPure diets (PC0.4 and PC0.8), a small amount of cocoa powder was used to double or to quadruple the polyphenol content of the conventional 10% cocoa diet (PC0.2). As about 50% of CocoaPure corresponded to polyphenols, the PC0.8 diet contained only 0.23% cocoa proteins, 0.16% cocoa carbohydrates, 0.11% cocoa lipids and 0.35% cocoa fiber, whereas the PC0.4 diet contained half proportions of these nutrients. Therefore, in the present study, three cocoa diets with increasing proportions of polyphenols (0.2%–0.8%) and decreasing content of other cocoa compounds, such as fiber, were designed to dissect the effects of cocoa polyphenols on the immune system.

During the study, all the rats fed the cocoa polyphenol-enriched diets had similar chow intakes to the reference animals. However, the animals in the PC0.2 group had lower body weight increase than the reference group during the study. These results are similar to those previously shown in high-dose cocoa-fed rats [17,21]. In this regard, flavonoids seem to stimulate cellular energy [29,30], cocoa intake is capable of inhibiting the lipid digestion and absorption [31,32,33] or of reducing fat deposition [33,34,35,36]. However, in the present study, the conventional cocoa diet (PC0.2), which provided the lowest amount of polyphenols, was the diet with the strongest effect on body weight. This result suggests that other compounds present in cocoa, such as fiber might also contribute to the inhibitory potency of the polyphenols [37]. Additionally the kind of flavonoids (monomeric, oligomeric or polymeric flavanols) contained in cocoa materials could also play a role in this effect on body weight increase. Another possible mechanism involved in the cocoa’s ability to reduce body weight and in lipid metabolism may be the polyphenols’ effect on microbiota composition. Differential effects of gut bacteria on the efficiency of energy extraction from the diet and changes in the host metabolism of absorbed calories have been described [38,39] and, for example, a high *Firmicutes*-to-*Bacteroidetes* ratio might have an important role in obesity [39,40,41]. In this sense, previous studies have demonstrated that cocoa reduces the *Clostridium* and *Staphylococcus* genera belonging to the *Firmicutes* phylum in the gut microbiota [19]. Although in the present study changes in microbiota composition were not evaluated, a reduction in the proportion of bacteria in feces was found in all the animals fed with cocoa polyphenol diets whereas no significant change in fecal pH was observed. This effect could be attributed to the antimicrobial activity of polyphenols, which has been reported both *in vitro* [42,43,44] and in experimental animals [45,46]. Focusing on cocoa polyphenols, our findings regarding the decreasing percentage of total bacteria seem to be in accordance with a previous study showing that polyphenols present in cocoa can produce an inhibitory effect on the growth of certain gut microbiota in rats [19]. Nevertheless, other studies have shown the ability of certain flavonoids present in cocoa to promote the growth of selected bacteria genera [47,48]. In any case, further studies should be carried out to look in detail at the effects of cocoa polyphenols on the microbiota composition and metabolism.

Regarding the systemic humoral response, previous studies have shown that immunoglobulin production is attenuated by experimental cocoa-enriched diets. Thus, splenocytes from young rats fed cocoa had a lower capacity to produce IgG, IgM and IgA and cocoa-fed rats had decreased serum IgG, IgM and IgA concentrations [17,18]. However, in the present study carried out in older rats for two weeks, all cocoa polyphenol diets tested had a tendency to lower the age-increasing pattern observed in the reference group but without significant differences and lacking a polyphenol dose-dependent effect. Therefore, it can be concluded that when the cocoa diet begins later in life and it lasts just two weeks, the immunomodulating effect is not as clear as in early age. We cannot discard the idea that a longer exposure to cocoa diet might have a more significant effect. A lack of or low effect has also been described when the cocoa proportion included in the diet was lower [28].

Contrary to what happened in the systemic compartment, the suppressing effect of cocoa polyphenols on antibody concentration was higher in the intestinal compartment. Our results from a defatted conventional cocoa diet (PC0.2) confirmed the down-modulatory effect on intestinal IgA content shown in previous studies, including varied proportions of cocoa diets (2%–10%), different ages at the beginning of the dietary intervention (3–6 weeks old) and length of diet (3–9 weeks) [17,18,28]. In the same way, the PC0.4 and PC0.8 diets, with a higher polyphenol content than the above-mentioned diet, also drastically decreased the IgA production but without a polyphenol dose-dependent effect. The effect of cocoa on intestinal IgA could be due, as has been described, to the influence of cocoa on genes related to Th maturation, Th-B cell interactions, and IgA+ B cell gut homing, among others [18,28]. Regardless of the cocoa-induced mechanisms, antibody-attenuating effects have also been previously described for different dietary polyphenols such as genistein [49], luteolin [50] and apigenin [51] in healthy or hypersensitivity animal models.

With regards to IgA-coating bacteria, similar results to the intestinal IgA content were found. The lowering effect of a defatted cocoa polyphenol diet (PC0.2) on IgA-coating bacteria in old animals agrees with previous studies performed on younger animals [19]. Again, there was a lack of correlation between the total polyphenol content in the diet and IgA-modulating effects, which means that cocoa diets based on higher flavonoids/lower cocoa fiber (CocoaPure) displayed similar effects. Therefore, the intestinal IgA down-modulatory effects described here could be partially attributed to cocoa polyphenols, because all diets, even with a very low cocoa fiber content (PC0.4 and PC0.8), were able to modulate the intestinal IgA production. However, a particular effect according to the kind of flavanols found on conventional or unfermented cocoa matter cannot be discarded.

In any case, a high whole cocoa or cocoa polyphenol intake might play an immunoregulatory role that could be beneficial on the hypersensitivity status. The immunomodulatory ability of cocoa can be especially significant in diseases involving a dysregulation of intestinal antibody responses such as celiac disease or food allergies.

## 5. Conclusions

The immunomodulatory effects of diets containing high cocoa could be partially attributed to its polyphenol content. However, other cocoa compounds and the particular polyphenol composition could also be key factors in this effect. Further studies evaluating dissected components of cocoa, such as individual polyphenols, fiber, theobromine, *etc.* should be carried out in order to evaluate whether it is possible to reproduce, at least partially, the previously and herein described cocoa effects on the immune system.

## References

[1] Henderson J.S., Joyce R.A., Hall G.R., Hurst W.J., McGovern P.E. (2007). Chemical and archaeological evidence for the earliest cacao beverages. Proc. Natl. Acad. Sci. USA.

[2] Dillinger T.L., Barriga P., Escárcega S., Jimenez M., Salazar L.D., Grivetti L.E. (2000). Food of the Gods: Cure for humanity? A cultural history of the medicinal and ritual use of chocolate. J. Nutr..

[3] Vinson J.A., Proch J., Zubik L. (1999). Phenol antioxidant quantity and quality in foods: Cocoa, dark chocolate and milk chocolate. J. Agric. Food Chem..

[4] Nishimoto N., Kishimoto T., Yoshizaki K. (2000). Anti-interleukin 6 receptor antibody treatment in rheumatic disease. Ann. Rheum. Dis..

[5] Hammerstone J.F., Lazarus S.A., Mitchell A.E., Rucker R., Schmitz H.H. (1999). Identification of procyanidins in cocoa (*Theobroma cacao*) and chocolate using high-performance liquid chromatography/mass spectrometry. J. Agric. Food Chem..

[6] Pietta P.G. (2000). Flavonoids as antioxidants. J. Nat. Prod..

[7] Lee K.W., Kim Y.J., Lee H.J., Lee C.Y. (2003). Cocoa has more phenolic phytochemicals and a higher antioxidant capacity than teas and red wine. J. Agric. Food Chem..

[8] Visioli F., Bernaert H., Corti R., Ferri C., Heptinstall S., Molinari E., Poli A., Serafini M., Smit H.J., Vinson J.A. (2009). Chocolate, lifestyle, and health. Crit. Rev. Food Sci. Nutr..

[9] Gu L., House S.E., Wu X., Ou B., Prior R.L. (2006). Procyanidin and catechin contents and antioxidant capacity of cocoa and chocolate products. J. Agric. Food Chem..

[10] Andrés-Lacueva C., Monagas M., Khan N., Izquierdo-Pulido M., Urpi-Sarda M., Permanyer J., Lamuela-Raventós R.M. (2008). Flavanol and flavonol contents of cocoa powder products: Influence of the manufacturing process. J. Agric. Food Chem..

[11] Wollgast J., Anklam E. (2000). Review on polyphenols in *Theobroma cacao*: Changes in composition during the manufacture of chocolate and methodology for identification and quantification. Food Res. Int..

[12] Sánchez-Rabaneda F., Jáuregui O., Casals I., Andrés-Lacueva C., Izquierdo-Pulido M., Lamuela-Raventós R.M. (2003). Liquid chromatographic/electrospray ionization tandem mass spectrometric study of the phenolic composition of cocoa (*Theobroma cacao*). J. Mass Spectrom..

[13] Lazarus S.A., Hammerstone J.F., Schmitz H.H. (1999). Chocolate contains additional flavonoids not found in tea. Lancet.

[14] Ramiro-Puig E., Casadesús G., Lee H.G., Zhu X., McShea A., Perry G., Pérez-Cano F.J., Smith M.A., Castell M. (2009). Neuroprotective effect of cocoa flavonoids on in vitro oxidative stress. Eur. J. Nutr..

[15] Martín M.A., Granado-Serrano A.B., Ramos S., Pulido M.I., Bravo L., Goya L. (2010). Cocoa flavonoids up-regulate antioxidant enzyme activity via the ERK1/2 pathway to protect against oxidative stress-induced apoptosis in HepG2 cells. J. Nutr. Biochem..

[16] Kenny T.P., Shu S.A., Moritoki Y., Keen C.L., Gershwin M.E. (2009). Cocoa flavanols and procyanidins can modulate the lipopolysaccharide activation of polymorphonuclear cells *in vitro*. J. Med. Food.

[17] Ramiro-Puig E., Pérez-Cano F.J., Ramírez-Santana C., Castellote C., Izquierdo-Pulido M., Permanyer J., Franch A., Castell M. (2007). Spleen lymphocyte function modulated by a cocoa-enriched diet. Clin. Exp. Immunol..

[18] Pérez-Berezo T., Franch A., Castellote C., Castell M., Pérez-Cano F.J. (2012). Mechanisms involved in down-regulation of intestinal IgA in rats by high cocoa. J. Nutr. Biochem..

[19] Massot-Cladera M., Pérez-Berezo T., Franch A., Castell M., Pérez-Cano F.J. (2012). Cocoa modulatory effect on rat fecal microbiota and colonic crosstalk. Arch. Biochem. Biophys..

[20] Ramiro-Puig E., Pérez-Cano F.J., Ramos-Romero S., Pérez-Berezo T., Castellote C., Permanyer J., Franch A., Izquierdo-Pulido M., Castell M. (2008). Intestinal immune system of young rats influenced by cocoa-enriched diet. J. Nutr. Biochem..

[21] Pérez-Berezo T., Ramiro-Puig E., Pérez-Cano F.J., Castellote C., Permanyer J., Franch A., Castell M. (2009). Influence of a cocoa-enriched diet on specific immune response in ovalbumin-sensitized rats. Mol. Nutr. Food Res..

[22] Ramos-Romero S., Pérez-Cano F.J., Castellote C., Castell M., Franch A. (2012). Effect of cocoa-enriched diets on lymphocytes involved in adjuvant arthritis in rats. Br. J. Nutr..

[23] Niemenak N., Rohsius C., Elwers S., Ndoumou D.O., Lieberei R. (2006). Comparative study of different cocoa (*Theobroma cacao* L.) clones in terms of their phenolics and anthocyanins contents. J. Food Compos. Anal..

[24] Tomas-Barberán F.A., Cienfuegos-Jovellanos E., Marín A., Muguerza B., Gil-Izquierdo A., Cerda B., Zafrilla P., Morillas J., Mulero J., Ibarra A. (2007). A new process to develop a cocoa poder with higher flavonoid monomer content and enhanced bioavailability in healthy humans. J. Agric. Food Chem..

[25] Cienfuentes-Jovellanos E., Pasamar M.A., Fritz J., Arcos J., Ramón D., Castilla Y. (2007). Method for Obtaining Polyphenol-Rich Cocoa Powder with a Low Fat Content and Cocoa thus Obtained. Patent Cooperation Treaty (PCT).

[26] Serra Bonvehí J., Ventura Coll F. (1997). Evaluation of bitterness and astringency of polyphenolic compounds in cocoa powder. Food Chem..

[27] Cienfuegos-Jovellanos E., Quiñones Mdel M., Muguerza B., Moulay L., Miguel M., Aleixandre A. (2009). Antihypertensive effect of a polyphenol-rich cocoa poder industrially processed to preserve the original flavonoids of the cocoa beans. J. Agric. Food Chem..

[28] Pérez-Berezo T., Franch A., Ramos-Romero S., Castellote C., Pérez-Cano F.J., Castell M. (2011). Cocoa-enriched diets modulate intestinal and systemic humoral immune response in young adult rats. Mol. Nutr. Food Res..

[29] Matsui N., Ito R., Nishimura E., Yoshikawa M., Kato M., Kamei M., Shibata H., Matsumoto I., Abe K., Hashizume S. (2005). Ingested cocoa can prevent high-fat diet-induced obesity by regulating the expression of genes for fatty acid metabolism. Nutrition.

[30] Nogueira L., Ramirez-Sanchez I., Perkins G.A., Murphy A., Taub P.R., Ceballos G., Villarreal F.J., Hogan M.C., Malek M.H. (2011). (–)-Epicatechin enhances fatigue resistance and oxidative capacity in mouse muscle. J. Physiol..

[31] Gu Y., Hurst W.J., Stuart D.A., Lambert J.D. (2011). Inhibition of key digestive enzymes by cocoa extracts and procyanidins. J. Agric. Food Chem..

[32] Gu Y., Yu S., Lambert J.D. (2013). Dietary cocoa ameliorates obesity-related inflammation in high fat-fed mice. Eur. J. Nutr..

[33] Min S.Y., Yang H., Seo S.G., Shin S.H., Chung M.Y., Kim J., Lee S.J., Lee K.W. (2013). Cocoa polyphenols suppress adipogenesis *in vitro* and obesity *in vivo* by targeting insulin receptor. Int. J. Obes. (Lond.).

[34] Lin J.K., Lin-Shiau S.Y. (2006). Mechanisms of hypolipidemic and anti-obesity effects of tea and tea polyphenols. Mol. Nutr. Food Res..

[35] Cherniack E.P. (2011). Polyphenols: Planting the seeds of treatment for the metabolic syndrome. Nutrition.

[36] Shin S.K., Ha T.Y., McGregor R.A., Choi M.S. (2011). Long-term curcumin administration protects against atherosclerosis via hepatic regulation of lipoprotein cholesterol metabolism. Mol. Nutr. Food Res..

[37] Ramos S., Moulay L., Granado-Serrano A.B., Vilanova O., Muguerza B., Goya L., Bravo L. (2008). Hypolipidemic effect in cholesterol-fed rats of a soluble fiber-rich product obtained from cocoa husks. J. Agric. Food Chem..

[38] Tumbaugh P.J., Bäckhed F., Fulton L., Gordon J.I. (2008). Diet-induced obesity is linked to marked but reversible alterations in the mouse distal gut microbiome. Cell Host Microbe.

[39] Tagliabue A., Elli M. (2013). The role of gut microbiota in human obesity: Recent findings and future perspectives. Nutr. Metab. Cardiovasc. Dis..

[40] Sanz Y., Rastmanesh R., Agostonic C. (2013). Understanding the role of gut microbes and probiotics in obesity: How far are we?. Pharmacol. Res..

[41] Shen J., Obin M.S., Zhao L. (2013). The gut microbiota, obesity and insulin resistance. Mol. Aspects Med..

[42] Puupponen-Pimiä R., Nohynek L., Hartmann-Schmidlin S., Kähkönen M., Heinonen M., Määttä-Riihinen K., Oksman-Caldentey K.M. (2005). Berry phenolics selectively inhibit the growth of intestinal pathogens. J. Appl. Microbiol..

[43] Lee H.C., Jenner A.M., Low C.S., Lee Y.K. (2006). Effect of tea phenolic and their aromatic fecal bacterial metabolites on intestinal microbiota. Res. Microbiol..

[44] Almajano M.P., Carbó R., Jiménez J.A.L., Gordon M.H. (2008). Antioxidant and antimicrobial activities of tea infusions. Food Chem..

[45] Dolara P., Luceri C., de Filippo C., Femia A.P., Giovannelli L., Caderni G., Cecchini C., Silvi S., Orpianesi C., Cresci A. (2005). Red wine polyphenols influence carcinogenesis, intestinal microflora, oxidative damage and gene expression profiles of colonic mucosa in F344 rats. Mutat. Res..

[46] Molan A.L., Liu Z., Tiwari R. (2010). The ability of green tea to positively modulate key markers of gastrointestinal function in rats. Phytother. Res..

[47] Tzounis X., Rodriguez-Mateos A., Vulevic J., Gibson G.R., Kwik-Uribe C., Spencer J.P. (2011). Prebiotic evaluation of cocoa-derived flavanols in healthy humans by using a randomized, controlled, double-blind, crossover intervention study. Am. J. Clin. Nutr..

[48] Fogliano V., Corollaro M.L., Vitaglione P., Napolitano A., Ferracane R., Travaglia F., Arlorio M., Costabile A., Klinder A., Gibson G. (2011). *In vitro* bioaccessibility and gut biotransformation of polyphenols present in the water-insoluble cocoa fraction. Mol. Nutr. Food Res..

[49] Kogiso M., Sakai T., Mitsuya K., Komatsu T., Yamamoto S. (2006). Genistein suppresses anti-specific immune responses through competition with 17β-estradiol for estrogen receptors in ovalbumin immunized BALB/c mice. Nutrition.

[50] Das M., Ram A., Ghosh B. (2003). Luteolin alleviated bronchoconstriction and airway hyperreactivity in ovalbumin sensitized mice. Inflamm. Res..

[51] Yano S., Umeda D., Yamashita S., Yamada K., Tachibana H. (2009). Dietary apigenin attenuates the development of atopic dermatitis-like skin lesions in NC/Nga mice. J. Nutr. Biochem..

